# Technical Modifications Employed in RARP to Improve Early Continence Recovery: A Literature Review

**DOI:** 10.3390/life15030415

**Published:** 2025-03-07

**Authors:** Ernesto Di Mauro, Roberto La Rocca, Francesco Di Bello, Ugo Amicuzi, Pasquale Reccia, Luigi De Luca, Francesco Paolo Calace, Michelangelo Olivetta, Gennaro Mattiello, Pietro Saldutto, Pierluigi Russo, Lorenzo Romano, Lorenzo Spirito, Carmine Sciorio, Biagio Barone, Felice Crocetto, Francesco Mastrangelo, Giuseppe Celentano, Antonio Tufano, Luigi Napolitano, Vincenzo Maria Altieri

**Affiliations:** 1 Department of Neuroscience, Reproductive and Odontostomatological Sciences, University of Naples “Federico II”, Via Pansini, 80138 Naples, Italy; ernestodm9@gmail.com (E.D.M.); robertolarocca87@gmail.com (R.L.R.); fran.dibello12@gmail.com (F.D.B.); u.amicuzi@gmail.com (U.A.); fellce.crocetto@gmail.com (F.C.); f.mastrangelo91@gmail.com (F.M.); 2 Urology Unit, AORN Ospedali dei Colli, Monaldi Hospital, 80131 Naples, Italy; reccia.pasquale1@gmail.com (P.R.); frap.calace@gmail.com (F.P.C.); 3 Division of Urology, Department of Surgical Multispecialty, AORN Antonio Cardarelli, 80131 Naples, Italy; luigideluca86@gmail.com; 4 Urology Unit, Gaetano Fucito Hospital, AOU San Giovanni di Dio e Ruggi d’Aragona, 84085 Salerno, Italy; olivetta.drmichelangelo@gmaii.com; 5 Division of Urology, Department of Surgical Sciences, AORN Sant’Anna e San Sebastiano, 81100 Caserta, Italy; drmattiellogennaro@gmail.com; 6 Department of Urology, Humanitas Gavazzeni, 24125 Bergamo, Italy; pietro.saldutto@gavazzeni.it; 7 Department of Urology, Fondazione Policlinico Universitario A. Gemelli, IRCCS, 00168 Rome, Italy; pierluigi.russo1@policlinicogemeili.it; 8 Unit of Urology, Department of Woman, Child and General and Specialized Surgery, University of Campania “Luigi Vanvitelli”, 80131 Naples, Italy; loryromano@hotmail.it (L.R.); lorenzospirito@msn.com (L.S.); 9 Urology Unit, Ospedale Alessandro Manzoni, 23900 Lecco, Italy; carmine.sciorio@gmail.com; 10 Department of Urology, ASL NA1 Centro Ospedale del Mare, 80147 Naples, Italy; biagio193@gmall.com; 11 IRCCS Neuromed, 86077 Pozzilli, Italy; dr.giuseppecelentano@gmail.com; 12 Department of Maternal Infant and Urologic Sciences, Policlinico Umberto I Hospital, “Sapienza” University of Rome, 00155 Rome, Italy; antonio.tufano91@gmail.com; 13 Azienda Sanitaria Locale (ASL)—Salerno DS66, Via Michele Vernieri, 84125 Salerno, Italy; 14 Department of Medicine and Health Sciences “V. Tiberio”, University of Molise, 86100 Campobasso, Italy; vincenzomaria.altieri@gmail.com

**Keywords:** prostatectomy, radical prostatectomy, robot-assisted radical prostatectomy, prostate surgery

## Abstract

Prostate cancer presents a substantial challenge, necessitating a delicate balance between effective treatment and preserving the overall quality of life for men, while robot-assisted radical prostatectomy (RARP) stands as the premier surgical approach, with a negligible rate of patients who remained incontinent. This review explores various technical modifications employed in RARP to improve early continence recovery, offering a summary of their implementation and potential benefits. Techniques like bladder neck preservation, subapical urethral dissection, and nerve-sparing approaches are critically discussed, highlighting their role in minimizing continence issues and ensuring a better post-operative experience for patients with prostate cancer.

## 1. Introduction

Prostate cancer (PCa) stands as the most prevalent cancer among males in numerous global regions and ranks as the second leading cause of cancer-related mortality in Europe and the United States of America [1]. It accounts for 7.3% of all new cancer cases in 2022, with 1.47 million new cases and approximately 396,800 deaths worldwide [2]. Despite the widespread adoption of prostate-specific antigen (PSA)-based screening, leading to a substantial increase in the diagnosis of low-risk PCa cases (cT1-2a, PSA < 10 ng/mL, ISUP grade 1), approximately 10% of patients continue to manifest locally advanced symptoms (such as bone pain, bilateral hydronephrosis, or acute urinary retention), posing a potentially life-threatening course [3]. PCa is an indolent disease with a non-negligible number of patients in whom the cancer evolutive, leading to metastasis and thus mortality [3,4]. The optimal management strategy for patients with locally advanced PCa currently lacks consensus [4]. However, reports highlight the use of laparoscopic or open radical prostatectomy (RP) as a major treatment option or as a part of a multimodal therapeutic strategy for locally advanced PCa, often complemented by additional interventions such as radiation therapy (RT) and androgen deprivation therapy (ADT) [5,6]. Retrospective research has shown that cancer-specific survival (CSS) rates can reach over 60% at 15 years, while overall survival (OS) rates can exceed 75% at 10 years [7]. This is impressive considering the increased likelihood of biochemical recurrence and the necessity for additional treatments [5,8]. Additionally, the PCa cohort studies have shown that even in situations of cT3b-T4 diseases, the 10-year CSS is more than 87% and the OS rate is 65% [9,10,11]. The European Association of Urology (EAU) guidelines now endorse the role of RP, as a viable treatment option for selective patients with advanced PCa, advocating its incorporation as one facet of a multimodal approach to care [4]. Like RP, RARP has emerged as the primary removal treatment, acknowledged as the gold standard [12]. Recent comparative studies of RARP and open radical prostatectomy have demonstrated similar superior rates of positive surgical margins and biochemical recurrence, as well as reduced need for blood transfusion and less blood loss [13,14]. However, despite the superiority of RARP over ORP a small fraction of PCA patients may experience urinary incontinence, negatively being affected in the quality of life (QoL) and psychological well-being of patients, regardless of their oncologic and sexual function outcomes [15]. Although over 80% of men achieve urine continence (defined as not needing to use pads) within the first year after surgery [16], a larger proportion will regain continence within two years following the treatment [17]. It is worth mentioning that the rates of early urinary continence are not very favorable. Indeed, the likelihood of a patient needing pads following surgery frequently varies from 70% to 80% at six weeks, 40% to 60% at three months, and 20% to 40% at six months, even when under the supervision of exceptionally skilled surgeons [18]. Therefore, several technical modifications have been suggested to enhance urinary continence in the context of RARP, including approaches such as preservation of bladder neck [19], hood approach, posterior and anterior reconstructions, subapical urethral dissection [20], as well as Retzius-sparing techniques and nerve-sparing methods [21]. This article presents a comprehensive analysis of the current body of literature, providing a concise overview of the surgical methods used in RARP to facilitate early restoration of continence

## 2. Materials and Methods

A narrative review of the literature was conducted to examine the surgical methods used in RARP to facilitate early restoration of continence. A systematic search was performed in three databases—PubMed^®^, Web of Science™, and Scopus^®^—in January 2024, with no chronological restrictions. The search strategy followed a free-text protocol using various combinations of the following keywords: “prostate cancer”, “prostatectomy”, “robotic”, “urinary continence”, and “incontinence”. The inclusion criteria encompassed retrospective and prospective studies (both comparative and non-comparative) narrative reviews, systematic reviews, and meta-analyses. Studies were selected based on their relevance, study design, sample size, and level of evidence. Exclusion criteria included letters, editorial comments, author replies, case reports, meeting abstracts, non-human studies, and non-English language publications. Study selection followed a two-step process. First, three senior authors (L.N., P.R., and R.L.R.) independently screened titles and abstracts to identify eligible studies. Full texts of the shortlisted articles were then assessed for inclusion based on predefined criteria. Any discrepancies were resolved through discussion among all authors. Additionally, reference lists of included studies were manually screened to identify further relevant articles.

## 3. Discussion

### 3.1. Bladder Neck Preservation (BNP)

Several approaches have been proposed to protect the circular fibers of the bladder neck in RARP with the aim of maintaining urinary continence [22]. The most used dissection planes include anterior, lateral, and anterolateral [22]. The bladder neck sparing technique provides greater sparing of the bladder neck. Neck preparation began with a careful and gentle dissection of the muscle fibers of the bladder wall. After detaching the superficial part, increasing the optical magnification can delicately dissect the urethra from the bladder neck. This technique allows us to create spaces on both sides of the bladder neck and preserve it. Subsequently, the posterior part of the urethra is mobilized and the Denonvilliers fascia is accessed. On this plane, the vas deferens and seminal vesicles are identified and isolated bilaterally. After having freed the prostate posteriorly, the prostatic apex is dissected, preserving the urethra in its entire length. Hemostasis is performed. After the removal of the prostate with the preservation of two vascular nerve bundles, proceed with reconstruction of the posterior plane and cervicourethral anastomosis. Regardless of the specific method employed, there is a unanimous agreement to safeguard the bladder neck at the highest possible level to facilitate early recovery of continence. Klein first proposed in 1992 the BNP method to accelerate continence recovery during RP and promote early return of urinary continence and erectile function. Deliveliotis et al. [23] were the pioneers in reporting cases of BNP that led to early recovery in continence rates in patients undergoing open RP. The strategy of BNP in RARP was first described by Freire et al. [24]. They conducted a study on 347 individuals who received the BNP technique and compared them to 271 patients who had the traditional RARP procedure. They found that the continence rates were much higher at 4 and 12 months for the patients who underwent the BNP technique [24]. Similarly, a study was conducted by Hashimoto et al. to identify factors that predict continence in patients who underwent RARP with bladder neck preservation (BNP). The study revealed a notable correlation between BNP and early continence [25]. Additionally, according to a recent meta-analysis, individuals who received RARP with BNP saw significant improvement in urinary continence outcomes at three to four months in comparison with those who underwent RARP surgical approach without BNP. The odds ratios (OR) were 2.88 (95% confidence interval at 3–4 months, 2.03 at 12 months, and 3.23 at 24 months) after RARP [26]. However, the debate over the potential increased risk of positive surgical margins (PSM) associated with BNP remains unsettled. According to the previous research, no significant difference was observed in the overall PSM rate or the PSM rate at the prostate base between the BNP and non-BNP groups [27]. A recently introduced technique in BNP is complete urethral sparing in which, without central zone tumors, the intraprostatic urethra is well-preserved. In this method, the bladder neck is not surgically cut until it reaches the verumontanum, which is the area where the urethra is usually narrower. This enables the establishment of a urethra–urethral anastomosis rather than the anastomosis between the bladder and the urethra. Via this surgical approach, initial functional and oncological outcomes have shown promising potential, with reported rates of immediate continence exceeding 50% after catheter removal [28].

### 3.2. Hood Technique

In 2021, Tewari and colleagues introduced a unique technique for RARP, known as the “hood technique” [28]. This approach focuses on preserving periurethral anatomical structures within the space of Retzius while sparing the pouch of Douglas [27]. This method involves the preservation of the contents within the Retzius area located in front of the prostate. As a result, the tissue that remains after the removal of the prostate takes on the appearance of a “hood” [21]. This anatomical hood encompasses certain fibers of the detrusor muscle, anterior vessels, puboprostatic ligament, arcus tendineus, and detrusor apron. The hood functions to encircle and safeguard the external sphincter, supportive structures and membranous urethra [22]. According to the research, the “hood technique” resulted in favorable continence results for patients, with a success rate of over 80% four weeks after the removal of the catheter [23]. By the 48-week post-catheter milestone, the continence rate further increased to an impressive 95%. Notably, this technique exhibited a low rate of positive surgical margins (PSM), standing at 6% [21]. In another study, the RARP “hood technique” was implemented to safeguard critical anatomical structures, such as the detrusor apron, puboprostatic ligament complex, arcus tendineus, endopelvic fascia, and pouch of Douglas [28]. The study reported continence rates at various time points after catheter removal, revealing a gradual improvement from 21% at 1 week to an impressive 95% at 48 weeks. The positive surgical margin rate was recorded at 6%, demonstrating a favorable outcome. Overall, according to this research, the hood technique exhibited promise in preserving musculofascial structures anterior to the urethral sphincter complex, facilitating early continence recovery without compromising positive surgical margin rates. The exclusion of anterior tumor locations contributed to a reduction in positive surgical margins. Additionally, complications were observed in 9.7% of patients, with the majority experiencing low-grade complications. However, this research was conducted within a single health system, which may impact the generalizability of the findings. Additionally, the study lacked randomization and a comparative arm.

### 3.3. Nerve Sparing Techniques

The traditional approach to nerve-sparing in RARP includes the separation of the NVBs from the posterolateral arc situated between the Denonvilliers’ and prostate fascia [29]. Numerous research has been conducted to investigate the anatomical localization of the nerves and improve functional outcomes after RARP, following Walsh and Donker’s explanation of the posterior and lateral course of the NVBs to the prostate. Recent investigations have shown that the path of NVBs is more diverse than previously thought, mainly located either in the posterolateral area or on the prostate anterior surface [30,31]. Additionally, the anterior nerve count rises from 6% to 11.2% as we move from the base to the apex [32]. As a result, a procedure called the Veil of Aphrodite, which involves a high nerve release technique, has been suggested as a way to enhance functional outcomes [33,34]. During this procedure, a surgeon performs dissection between the one o’clock and five o’clock positions on the right side and between the six o’clock and eleven o’clock positions on the left side, within the intrafascial/interfascial layers. Later, a modification was made to this technique, which proposed preserving tissue between the eleven o’clock and one o’clock positions, specifically the pubovesical ligaments [35]. Recognizing the increased complexity of this procedure (super veil), it is typically offered to patients in the low-risk group. Kaul et al. [33] conducted prospective research on 154 patients and found that 29 percent of subjects achieved continence upon catheter removal. Nevertheless, at the 12-month assessment, 97% of the patients had successfully attained continence, with a median duration of 14 days until they no longer required pads for urinary control. Therefore, they concluded that employing the Veil of Aphrodite approach could potentially speed up the achievement of continence at an earlier stage [33].

### 3.4. Subapical Urethral Dissection

The external sphincter is primarily located within the prostate, namely, between the verumontanum and apex [35,36]. This sphincter is partially covered by apical tissue due to anatomical changes in the morphology of the apex [37,38]. Hence, maintaining the complete urethral functional length also aids in the preservation of a portion of the external sphincter [37,38]. Mungovan et al. established a correlation between the preoperative measurement of urethral length using MRI and the early recovery of continence [39]. Specifically, they found that each additional millimeter of urethral length was related to a higher likelihood of immediate continence recovery. These results were further supported by Song et al., who demonstrated a strong correlation between the maximal length of the urethra before and after surgery and urine continence at 6- and 12-months following robot-assisted radical prostatectomy (RARP) [39]. The way to obtain a sufficiently long urethra without having positive margins is to correctly identify the border of the urethra with the prostatic apex. Tewari et al. [21] described in their work how to reduce the risk of positive margins, during this surgical procedure, by changing the 30° optic facing upwards with the 0 optic. The authors demonstrated that the best visualization given by the 0 optic involves a lower risk of positive surgical margins. Bianchi et al. [40] also described the “collar” technique for RARP to avoid positive surgical margins. This technique allows for the different anatomical layers of the prostatic urethra to be perfectly visualized. The external layers of the musculature are first dissected smooth, striated, and circular away from the prostate to avoid positive surgical margins. Subsequently the internal layer of the longitudinal smooth muscle is preserved in maximum extension and dissected close to the prostatic apex [3]. Additionally, the research conducted by Michl et al. showed that a careful apex dissection resulted in improved rates of urine continence in both the short-term and long-term, as compared to a broad excision [41]. In a recent study conducted by Hoeh et al., the implementation of complete functional-length urethral sphincter and neurovascular bundle (NVB) sparring in subjects having RARP led to a notable enhancement in long-term continence rates [42]. In fact, the rate of continence, which refers to the absence of urinary leakage or the usage of only one pad, reached 91% over a 12-month period [42].

### 3.5. Retzius-Sparing Technique

In 2010, Galfano et al. introduced Retzius-sparing robot-assisted radical prostatectomy (RARP), a type of posterior approach opening the prostate via the Douglas space [20]. The procedure involves a transverse incision at the peritoneal reflection beneath the rectovesical pouch, with subsequent recognition and organization of the seminal vesicles and vas deferens [20]. This procedure of antegrade dissection begins at the posterolateral and posterior prostate surfaces, with a lateral sweeping motion of the neurovascular bundles (NVBs) [20]. The bladder neck is thereafter separated, the dorsal venous complex is released via proper dissection, and the urethra is severed below the highest point, thus freeing the prostate. Retzius-sparing RARP preserves anterior anatomical structures, including the endopelvic fascia, puboprostatic ligament, DVC, pubovesical ligaments, and detrusor aprons [20]. This preservation provides support to the bladder anteriorly, contributing to early continence recovery. Galfano et al. reported early continence in over 90% of subjects, a finding supported by numerous subsequent publications [21,22,23,24,25,26,27,28,29,30,31,32,33,34,35,36,37,38,39,40,41,42,43,44,45,46].

Critics argue that the continence benefits of this technique diminish after six months when recovery rates become equal to the conventional approach. However, a 2020 meta-analysis indicated early better continence recovery with Retzius-sparing RARP up to 12 months [47]. A significant concern is the consistently reported higher positive surgical margin (PSM) rates in studies of Retzius-sparing RARP [48]. A metanalysis, systematic review, and MASTER study, incorporating four RCTs and six prospective observational studies, revealed statistically significantly higher PSM rates in ≤pT2 tumors following RS-RARP compared to conventional RARP [49]. A study conducted in Japan revealed that RS-RARP is linked to a greater rate of PSM in anterior tumors, whereas no such association was observed in posterior tumors when compared to traditional RARP [50]. The sparing of the detrusor apron and Santorini plexus likely reduces the distance between the resection plane and tumor edge, impacting PSM [49,50]. Additionally, when a surgeon considers RS-RARP, it is also important to recognize the steep learning curve necessary for the best results [49,50].

### 3.6. Posterior Retropubic Reconstruction

In the year 2001, Rocco and colleagues introduced their technique for posterior reconstruction as a component of open retropubic prostatectomy with the goal of enhancing continence recovery [51]. The Rocco stitch is made using a 16 cm × 16 cm double arm Quill 2.0 RB-1 thread. Posterior reconstruction is performed by bringing the prostatic vescico muscle to the medial raphe, using the suture, from a medial to lateral direction. The directionality of the point is to go from inside to outside in an inverted V shape [4] Posterior reconstruction serves two primary objectives: first, the approximation of Denonvilliers’ fascia to the posterior side of the posterior median raphe and rhabdosphincter, facilitating the cranial relocation of the urethral sphincter; second, it involves fixing Denonvilliers’ fascia to the posterior bladder wall [52]. This not only decreases tension in the anastomosis but also offers pelvic support to the bladder neck [50]. To ensure the technique’s success, it is crucial to meticulously preserve Denonvilliers’ fascia when dissecting between the rectum and prostate. Another crucial element of this method involves avoiding the lateral neurovascular bundles and urethra from the sutures used in the repair [52].

In 2007, Rocco et al. modified the approach for RARP, highlighting a substantial reduction in the time required for recovery of continence and illustrating the ease of using laparoscopic techniques [53]. Grasso et al. [54] performed a recent systematic review to evaluate the effects of PRR on incontinence, focusing on the surgical method used. Regarding RARP, the PRR approach exhibited a notable benefit in terms of both late and early continence, without any noticeable disparities in preoperative and postoperative problems linked to PR.

According to a survey performed by the European Association of Urology Robotic Urology Section (ERUS), 51.7% of the 116 surgeons’ surveys reported that they typically engage in posterior reconstruction, while 28.4% of them reported never incorporating this procedure into their practice [55].

### 3.7. Anterior Retropubic Suspension

The anterior retropubic suspension, also known as the Patel stitch, originated from Walsh’s description in open radical prostatectomy [56]. Subsequently, Patel et al. [56] implemented this methodology for RARP. The method involves the rhabdosphincter suspension in the pubic periosteum by using puboprostatic ligaments arranged in an eight-shape figure, following the ligation of the DVC. In their study encompassing 331 consecutive patients, Patel et al. [56] analyzed the results of 237 patients who underwent the suspension stitch procedure and compared them to the outcomes of 94 individuals who did not receive the stitch. The findings demonstrated a significant disparity in continence at three months following RARP (no need for pad, 83% vs. 92.8%), suggesting a faster recovery from incontinence when utilizing the suspension stitch. However, previous research found an important difference in the rate of complete continence (no need for pads) between individuals who had anterior reconstruction and those who did not, both at the start of the removal of the catheter and six months after undergoing RARP [57]. The researchers strongly recommended the implementation of a thorough anatomical reconstruction approach at their facility, which included both anterior and posterior reconstruction approaches [57].

## 4. Controversies and Future Perspective

There are still controversies about continence outcomes in patients after RP. Although several techniques have been described during the time there are multiple limitations as well as different definitions of continence, little data on technique, and very little randomized study. This is related to a difficulty in identifying the appropriate technique. Preservation strategies must take into account various anatomical and physiological factors, including the nerve supply to the urethra, vascular integrity, tissue support, prostate shape, and bladder position. Ultimately, the surgeon must determine which technique is both safe and oncologically sound. However, future advancements in both surgical approaches and technology may play an equally critical role in refining RP and improving continence outcomes [58]. While the retropubic approach remains the gold standard in robotic-assisted radical prostatectomy (RARP), alternative techniques such as lateral, transvesical, and transperineal approaches have gained interest. The lateral approach aims to preserve the levator ani muscle and periprostatic fascia, potentially reducing postoperative incontinence. The transvesical approach, which avoids direct manipulation of the dorsal venous complex and endopelvic fascia, has been proposed as a minimally invasive alternative, particularly in cases of previous pelvic surgery or narrow pelvis anatomy. The transperineal approach, although less commonly used in robotic surgery, provides direct access to the apical and posterior prostate, which may influence sphincter preservation and continence outcomes. However, further high-quality studies are needed to compare these approaches in terms of functional and oncological results. At the same time, the continuous evolution of robotic platforms, AI-assisted surgery, and real-time intraoperative imaging may enhance precision and allow for more individualized, nerve- and tissue-sparing approaches. Innovations such as augmented reality overlays, intraoperative neuromonitoring, and enhanced robotic haptic feedback may facilitate better preservation of functional structures critical for continence. Moreover, patient-specific 3D surgical planning and machine learning algorithms for predicting continence recovery could help tailor surgical strategies to each patient’s anatomical and functional characteristics. Postoperative management also remains a key area of development. While pelvic floor muscle exercises (PFME) continue to be the first-line conservative therapy, pharmacologic and surgical interventions are evolving. Duloxetine, though off-label, remains an option for temporary symptom relief, while new-generation artificial urinary sphincters (AUS) and male slings are under investigation to optimize treatment for post-prostatectomy incontinence. In conclusion, post RARP continence depends on multiple factors including preoperative patient and tumor factors and surgical factors. While a number of these factors are non-modifiable, there are several surgical techniques, which can improve continence post RARP (Table 1). Therefore, attention should be focused on developing and utilizing these techniques when performing RARP, especially in men with high risk of developing incontinence. The therapeutic decisions and the treatment options must be individualized for each patient according to clinical and social factors. With this perspective, the technological improvements and the emergence of new dedicated treatments and devices have created a continuously positive evolution of clinical outcomes in these patients. 

## 5. Strength and Limitations

The present study provides a comprehensive overview of the most widely adopted surgical techniques in RARP, synthesizing evidence from heterogeneous studies in a qualitative analysis that highlights both strengths and limitations of these approaches. The discussion acknowledges the variability in surgical strategies and the need for individualized decision-making based on patient anatomy, tumor characteristics, and surgeon expertise. However, certain limitations must be acknowledged. The available literature on this topic is often constrained by small sample sizes, retrospective designs, and short follow-up periods, with many studies originating from single-center experiences. Additionally, a direct comparative analysis between techniques remains limited, making it challenging to establish definitive superiority among different approaches. Another key limitation is the lack of data on the applicability of these techniques in more advanced prostate cancer stages, particularly in oligometastatic prostate cancer. Given the distinct oncological challenges in this setting, surgical priorities may shift from early continence recovery to achieving optimal tumor debulking and systemic disease control. Certain techniques discussed—such as bladder neck preservation, nerve-sparing RARP, and Retzius-sparing RARP—may offer benefits in localized disease but may be less suitable for patients with metastatic involvement of the bladder base, lymph nodes, or anterior prostate. Conversely, standard RARP with precise resection techniques and reconstructive approaches may offer a better balance between oncologic safety and functional recovery in oligometastatic prostate cancer patients. Lastly, real-world surgical decision-making is inherently dynamic, influenced by patient-specific factors and surgeon preferences. The customization of RARP techniques in clinical practice may differ from standardized study protocols, further underscoring the importance of individualized surgical planning. Future research, particularly prospective and comparative studies, is necessary to refine surgical strategies in both localized and oligometastatic disease settings. 

## 6. Conclusions

Several surgical techniques exist within RARP to boost urinary continence recovery following PCa treatment. Despite each method showing different advantages and drawbacks, the continence post RARP depends on different factors, i.e., patient, tumor, and surgical factors. Furthermore, several studies with a large sample size are necessary to better develop this technique and identify the patients to improve continence post RARP.

## Figures and Tables

**Table 1:** Characteristics of studies included in this review.

Authors, Year	Sample Size	Technique	Early Continence Rate	Late Continence Rate	Oncological Outcames
Deliveliotis, 2002 [23]	48	BNP	n.a.	92%	No statistically significant difference
	51	PLS	n.a.	92%	
	50	Both previous techinque	n.a.	94%	
Freire, 2009 [24]	348	BNP	65.6%	100%	No statistically significant difference
Wagaskar, 2020 [28]	300	HT	88%	95%	n.a.
Patel, 2009 [56]	237	Periurethral retropubic suspension stitch	92.8%	97.9%	n.a.
Rocco, 2001 [51]	24	Reconstruction of the urethral striated sphincter	79.2%	n.a.	n.a.
Puliatti, 2019 [57]	48	Anterior puboprostatic ligament reconstruction	56.3%	89.6%	n.a.

BNP: bladder neck preservation; PLS: puboprostatic ligament sparing; HT: hood technique.
